# Between Chemical Simplicity and Biological Complexity: In Silico Profiling of Butyrolactones I and III as Potential Multi-Target Drug Candidates

**DOI:** 10.3390/cimb48070700

**Published:** 2026-07-10

**Authors:** Tomasz Kowalczyk, Anna Merecz-Sadowska, Belma Konuklugil, İbrahim Seyda Uras, Radosław Zajdel, Patricia Rijo, Przemysław Sitarek

**Affiliations:** 1 Department of Molecular Biotechnology and Genetics, Faculty of Biology and Environmental Protection, University of Lodz, 90-237 Lodz, Poland; 2 Department of Economic and Medical Informatics, University of Lodz, 90-214 Lodz, Poland; anna.merecz-sadowska@uni.lodz.pl (A.M.-S.); radoslaw.zajdel@uni.lodz.pl (R.Z.); 3 Department of Pharmacognosy, Faculty of Pharmacy, Lokman Hekim University, 06530 Ankara, Turkey; belma.konuklugil@lokmanhekim.edu.tr; 4 Department of Pharmacognosy, Faculty of Pharmacy, Ağrı İbrahim Çeçen University, 04100 Agri, Turkey; isuras@agri.edu.tr; 5 Department of Intelligent Systems in Health Monitoring, Medical University of Lodz, 90-645 Lodz, Poland; 6 CBIOS-Research Center for Biosciences & Health Technologies, Universidade Lusófona, 1749-024 Lisbon, Portugal; patricia.rijo@ulusofona.pt; 7 iMed.ULisboa-Research Institute for Medicines, Faculdade de Farmácia da Universidade de Lisboa, Av. Prof. Gama Pinto, 1649-003 Lisbon, Portugal; 8 Department of Medical Biology, Medical University of Lodz, 90-151 Lodz, Poland

**Keywords:** butyrolactone I, butyrolactone III, *Aspergillus terreus*, ADMET, molecular docking, molecular dynamics, acetylcholinesterase, MDM2, multitarget drug discovery, natural products

## Abstract

The development of multi-targeted therapeutic agents is increasingly recognized as essential for treating multifactorial diseases. Butyrolactone I and butyrolactone III, γ-butyrolactone derivatives isolated from the marine fungus *Aspergillus terreus*, represent structurally related natural products with largely unexplored polypharmacological potential. This study employed a comprehensive in silico approach combining ADMET profiling, quantum chemical calculations, molecular docking, and molecular dynamics simulations to evaluate their therapeutic potential across multiple pharmacological targets. Physicochemical analysis revealed favorable drug-like properties for both compounds, with complete compliance with Lipinski’s Rule of Five, high predicted gastrointestinal absorption (>80%), and acceptable toxicity profiles (toxicity class 4, LD_50_ = 2000 mg/kg). Neither compound showed hepatotoxic, neurotoxic, cardiotoxic, carcinogenic, or mutagenic liabilities. Frontier molecular orbital analysis (DFT/B3LYP/6-31G(d,p)) revealed comparable HOMO energies (−6.054 and −6.059 eV), with butyrolactone III exhibiting enhanced kinetic stability based on a larger HOMO–LUMO gap (4.662 eV vs. 4.443 eV) and higher chemical hardness (η = 2.331 eV vs. 2.222 eV). Molecular docking against four therapeutic targets revealed target-selective binding profiles: butyrolactone III demonstrated binding affinity toward acetylcholinesterase exceeding donepezil (−9.0 vs. −8.3 kcal/mol), while butyrolactone I exhibited MDM2 binding affinity slightly exceeding nutlin-3a (−7.8 kcal/mol). Both compounds showed moderate interactions with COX-2 and topoisomerase IV. Molecular dynamics simulations validated the stability of AChE complexes (RMSD < 2.0 Å) and the MDM2–butyrolactone I complex (RMSD: 0.69 ± 0.09 Å), while the MDM2–butyrolactone III complex exhibited significant instability (RMSD up to 3.55 Å), highlighting the critical role of the prenyl group in MDM2 recognition. These findings, consistent with, though not a direct experimental validation of, previously published in vitro data, support the evaluation of butyrolactone III as a scaffold for neuroprotective agents and butyrolactone I as a p53 pathway modulator for cancer therapy, illustrating the potential value of fungal metabolites in multi-target drug discovery and the role of integrated computational approaches in prioritizing candidates for subsequent experimental testing.

## 1. Introduction

The rapid increase in the incidence of diseases with complex etiology, such as chronic inflammation, cancer, neurodegenerative disorders, and bacterial and fungal infections, represents one of the greatest challenges facing modern medicine [1,2]. The conventional approach to drug design, predicated on the “one drug–one target” paradigm, is increasingly inadequate, particularly in the context of mounting drug resistance and the multifactorial nature of numerous diseases [3]. Consequently, there is growing interest in multitarget agents and polypharmacological strategies capable of modulating several disease-relevant pathways simultaneously. In this context, natural products remain a highly valuable source of new chemical scaffolds with therapeutic potential [4,5,6]. Filamentous fungi, including hyphomycetes, are among the most prolific producers of structurally diverse secondary metabolites with broad biological activities [7].

The genus *Aspergillus* merits particular attention, especially *Aspergillus terreus*, which is distinguished by its capacity to synthesize numerous biologically active compounds, including polyketides, alkaloids, and lactones [8,9]. *A. terreus* is frequently associated with the synthesis of lovastatin, underscoring its significance as a source of substances with proven clinical applications [10,11]. In parallel, multiple reports indicate that secondary metabolites of this species exhibit anticancer, antimicrobial, anti-inflammatory, and neuroactive properties [12,13]. Among the compounds isolated from *A. terreus*, butyrolactone I and butyrolactone III, which belong to the γ-butyrolactone group, are of particular interest [14]. These compounds feature a rigid γ-lactone core decorated with aromatic substituents, a motif compatible with diverse noncovalent interactions and potentially enabling binding to multiple protein families [15]. Butyrolactone I has been demonstrated to possess anticancer properties, including the capacity to inhibit cyclin-dependent kinases (CDK) and regulate cell cycle progression [16,17]. However, a systematic, comparative evaluation of the broader biological space accessible to butyrolactones I and III, beyond the established CDK-related activity of butyrolactone I, remains limited [18,19,20]. Butyrolactones I and III were selected here as a closely matched structural pair differing essentially in a single moiety (an isoprenyl/prenyl group in butyrolactone I versus an epoxide in butyrolactone III), which makes them an informative model system for probing how this discrete chemical modification influences predicted physicochemical behavior, electronic structure, and target engagement.

In recent years, in silico methods have become an integral part of modern drug discovery and design strategies [21]. Specifically, techniques such as molecular docking, biological target prediction, pharmacophore modeling, and ADMET analysis enable rapid prioritization of compound–target hypotheses and early risk assessment [22,23]. Of particular significance is the ability of computational approaches to support analyses of polypharmacology, which is increasingly recognized as important for identifying compounds with multidirectional therapeutic effects [24,25]. The application of in silico methodologies to fungal secondary metabolites facilitates the identification of potential molecular targets associated with neoplasia, microbial survival, neurotransmission, and inflammation [26,27,28]. Accordingly, structure-based screening against representative targets (e.g., kinases, inflammatory mediators, neurotransmission-related proteins, and essential microbial enzymes) can reveal plausible mechanistic entry points for subsequent experimental validation [29,30].

Here, we provide an in silico, multi-parameter assessment of butyrolactone I and butyrolactone III as prospective natural-product-inspired drug candidates. Our workflow integrates predicted pharmacokinetic and toxicity (ADMET) profiles, electronic-structure descriptors relevant to reactivity and intermolecular recognition, molecular docking against a panel of pharmacologically distinct targets, and molecular dynamics simulations to assess the stability of the predicted binding modes. Together, these complementary analyses highlight potential opportunities and liabilities for both compounds, providing a rational basis for subsequent experimental validation and optimization within the framework of designing new multi-target drug candidates. To our knowledge, this is the first study to combine ADMET profiling, DFT-based electronic-structure analysis, molecular docking, and molecular dynamics in a single comparative polypharmacology assessment of butyrolactones I and III, and to exploit the prenyl-versus-epoxide difference between them as a structure–activity probe across several pharmacologically distinct target classes.

## 2. Materials and Methods

### 2.1. In Silico ADMET Prediction

The pharmacokinetic properties of butyrolactone I and butyrolactone III were predicted using the SwissADME web server (http://www.swissadme.ch/) (accessed on 15 April 2026) [31]. The canonical SMILES representations of both compounds were used as input. Physicochemical parameters including molecular weight, number of heavy atoms, hydrogen bond donors and acceptors, rotatable bonds, and topological polar surface area (TPSA) were calculated. Lipophilicity was assessed using multiple prediction methods (iLOGP, XLOGP3, WLOGP, MLOGP), and consensus Log *p* values were determined. Drug-likeness was evaluated according to Lipinski’s Rule of Five [32], Ghose filter [33], Veber rules [34], Egan rules [35], and Muegge criteria [36]. Pharmacokinetic properties including gastrointestinal (GI) absorption, blood–brain barrier (BBB) permeability, P-glycoprotein (P-gp) substrate status, and cytochrome P450 enzyme inhibition (CYP1A2, CYP2C9, CYP2C19, CYP2D6, CYP3A4) were predicted.

### 2.2. Toxicity Prediction

Toxicological profiles were evaluated using the ProTox-3.0 web server (https://tox.charite.de/protox3/) (accessed on 15 April 2026) [37]. The SMILES structures of both compounds were submitted for prediction of acute oral toxicity (LD_50_), toxicity class classification (according to the Globally Harmonized System), and organ-specific toxicity endpoints including hepatotoxicity, cardiotoxicity, nephrotoxicity, and neurotoxicity. Additional toxicological parameters such as mutagenicity, carcinogenicity, cytotoxicity, and immunotoxicity were also assessed.

### 2.3. Quantum Chemical Calculations

Density functional theory (DFT) calculations were performed using the ORCA 5.0 quantum chemistry program package [38]. The molecular geometries of butyrolactone I and butyrolactone III were optimized at the B3LYP/6-31G(d,p) level of theory [39,40]. Frequency calculations were performed to confirm that the optimized structures corresponded to true energy minima (no imaginary frequencies). Frontier molecular orbital (FMO) analysis was conducted to determine the energies of the highest occupied molecular orbital (HOMO) and lowest unoccupied molecular orbital (LUMO). Global chemical reactivity descriptors were calculated from HOMO and LUMO energies, including ionization potential (I = −E_HOMO), electron affinity (A = −E_LUMO), HOMO-LUMO energy gap (ΔE = E_LUMO − E_HOMO), chemical hardness (η = (I − A)/2), chemical softness (S = 1/(2η)), electronegativity (χ = (I + A)/2), chemical potential (μ = −χ), and electrophilicity index (ω = χ^2^/(2η)) [40,41].

### 2.4. Molecular Docking

Molecular docking studies were performed using AutoDock Vina version 1.2.5 [42] to evaluate the binding affinity of butyrolactone I and butyrolactone III against four therapeutic targets representing different pharmacological activities: anti-inflammatory (COX-2), antibacterial (Topoisomerase IV), anticancer (MDM2), and neuroprotective (AChE). Grid box parameters were determined based on the positions of co-crystallized reference ligands (Table 1). The four targets were deliberately chosen to represent four therapeutic areas previously associated with A. terreus secondary metabolites (inflammation, bacterial infection, cancer, and neurodegeneration) rather than to map a single shared disease pathway, reflecting the polypharmacology hypothesis under investigation. Each target is a well-characterized, druggable protein available as a high-resolution structure co-crystallized with a clinically or pharmacologically relevant ligand (donepezil for AChE, nutlin-3a for MDM2, rofecoxib for COX-2, and novobiocin for topoisomerase IV), allowing the docking grid to be centered on the experimentally defined binding site and the butyrolactone scores to be benchmarked against the corresponding reference inhibitor. Cyclin-dependent kinases, the experimentally established targets of butyrolactone I, were intentionally not included in this panel, since the aim was to explore previously uncharacterized target space for the two compounds; the known CDK activity is instead used as an orthogonal, literature-based reference point. Target selection followed a literature-guided, criterion-based procedure rather than an unbiased proteome-wide screen. Candidate targets were first compiled from published experimental and computational reports on butyrolactones and other *A. terreus* secondary metabolites across anti-inflammatory, antibacterial, anticancer, and neuroactive activities. From this pool, four targets were prioritized according to several criteria. The first was documented relevance of the corresponding therapeutic area to *A. terreus* metabolites. The second was the availability of a high-resolution crystal structure co-crystallized with a clinically or pharmacologically validated ligand, permitting reproducible grid definition and score benchmarking. The third was representation of mechanistically distinct protein families, namely an inflammatory oxygenase, a bacterial type II topoisomerase, a protein–protein interaction oncotarget, and a serine hydrolase, to probe polypharmacology across target space. The fourth was non-redundancy with the cyclin-dependent kinase activity already experimentally established for butyrolactone I. To verify the docking protocol, each co-crystallized reference ligand was re-docked into its binding site under identical settings; the predicted poses recovered the experimentally observed binding orientation, and the corresponding scores are reported alongside the butyrolactone values as an internal benchmark.

Protein structures were obtained from the Protein Data Bank (PDB) and prepared by removing water molecules and heteroatoms, followed by addition of polar hydrogens. Ligand structures were generated from SMILES notation using Open Babel [43], with 3D coordinate generation, assignment of protonation states corresponding to physiological pH (7.4), MMFF94 energy minimization, and Gasteiger partial charges. Docking was performed with exhaustiveness = 64 and num_modes = 20 to ensure thorough conformational sampling.

Grid box coordinates were determined by locating reference ligand centers in original crystal structures using PyMOL version 3.1.6.1. For COX-2 (5KIR), the grid box was centered at coordinates x = 23.2 Å, y = 1.3 Å, z = 34.3 Å with dimensions of 22 × 22 × 22 Å. For topoisomerase IV (1S14), the center was positioned at x = 38.1 Å, y = 43.9 Å, z = 54.3 Å with box dimensions of 22 × 22 × 22 Å. The MDM2 (4HG7) grid box was centered at x = −23.8 Å, y = 7.5 Å, z = −14.1 Å with dimensions of 24 × 24 × 24 Å. For AChE (4EY7), the grid center was located at x = −1.6 Å, y = −50.2 Å, z = 2.1 Å with box dimensions of 22 × 22 × 26 Å.

Three-dimensional visualization of protein–ligand complexes was performed using PyMOL 2.5 [44]. Proteins were displayed as gray cartoons, ligands as orange sticks, and binding site residues as yellow sticks. The binding pocket surface was rendered in transparent gray. Two-dimensional interaction diagrams illustrating hydrogen bonds, hydrophobic interactions, and π–π stacking were generated using BIOVIA Discovery Studio Visualizer 2021 [45].

### 2.5. Molecular Dynamics Simulations

Molecular dynamics (MD) simulations were performed to evaluate the stability of the docked complexes. The simulation systems were prepared using CHARMM-GUI version 1.7 Solution Builder (https://www.charmm-gui.org/) (accessed on 15 April 2026) [46]. The protein-ligand complexes obtained from molecular docking were solvated in a rectangular water box with TIP3P water molecules, maintaining a minimum distance of 10 Å between the solute and the box edges. The systems were neutralized and ionized with 0.15 M NaCl to mimic physiological conditions.

The CHARMM36m force field was employed for proteins [47], while ligand parameters were generated using the CHARMM General Force Field (CGenFF) through the CHARMM-GUI Ligand Reader & Modeler module [48]. All simulations were performed using NAMD 2.14 [49] with GPU acceleration. The simulation protocol consisted of the following steps: Energy minimization: 10,000 steps of conjugate gradient minimization; Equilibration: 125,000 steps (250 ps) under NVT ensemble with position restraints on protein backbone atoms; Production: 100 ns NPT ensemble simulation.

Production simulations were conducted at 310 K (37 °C) using Langevin dynamics for temperature control (damping coefficient: 1.0 ps^−1^) and the Langevin piston method for pressure control (target: 1.01325 bar). The SHAKE algorithm was applied to constrain all bonds involving hydrogen atoms, allowing a 2 fs time step. Long-range electrostatic interactions were calculated using the Particle Mesh Ewald (PME) method with a grid spacing of 1.0 Å. A cutoff distance of 12 Å was used for non-bonded interactions with a switching function starting at 10 Å.

Trajectory analysis was performed using VMD 1.9.4 [50]. Root mean square deviation (RMSD) of protein backbone atoms and root mean square fluctuation (RMSF) of Cα atoms were calculated to assess structural stability and residue flexibility, respectively. Radius of gyration (Rg) was computed to monitor protein compactness throughout the simulation. Hydrogen bond interactions between ligands and protein residues were analyzed with donor-acceptor distance cutoff of 3.5 Å and angle cutoff of 30°.

## 3. Results

### 3.1. Physicochemical Properties

**Figure 1:**
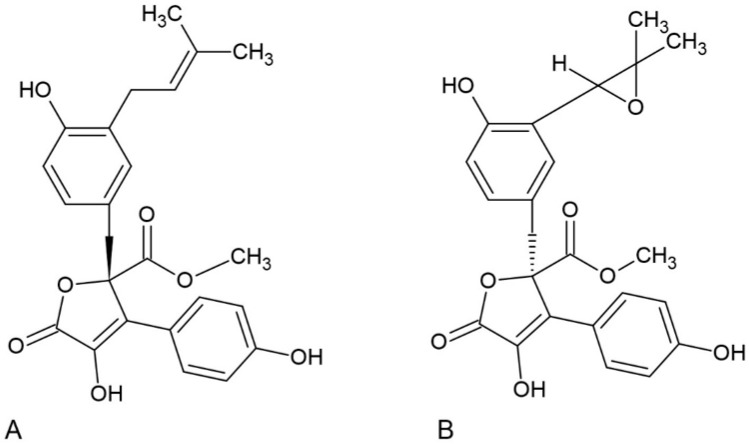
Structure formula of butyrolactone I (**A**) and III (**B**).

The physicochemical properties of both compounds were predicted using SwissADME. The molecular formulas are C_24_H_24_O_7_ for butyrolactone I and C_24_H_24_O_8_ for butyrolactone III, indicating that butyrolactone III contains an additional oxygen atom due to the presence of an epoxide group instead of the prenyl moiety (Figure 1). Both compounds exhibit similar physicochemical profiles with molecular weights within the acceptable range for oral drug candidates (<500 g/mol according to Lipinski’s rule).

The TPSA values of 113.29 Å^2^ and 125.82 Å^2^ for butyrolactone I and III respectively suggest moderate membrane permeability (Table 2). The number of hydrogen bond acceptors and donors is within acceptable limits for both compounds.

### 3.2. Lipophilicity and Solubility

Butyrolactone I exhibits slightly higher lipophilicity (Consensus Log *p* = 3.41) compared to butyrolactone III (Consensus Log *p* = 2.78). This difference can be attributed to the presence of the more lipophilic prenyl group in butyrolactone I versus the more polar epoxide moiety in butyrolactone III. Both compounds fall within the optimal lipophilicity range for oral drug absorption (Table 3).

According to ESOL and Silicos-IT predictions, both compounds are classified as moderately soluble in water. However, the Ali model predicts butyrolactone I to be poorly soluble, whereas butyrolactone III is moderately soluble. Butyrolactone III generally shows better aqueous solubility, which correlates with its lower lipophilicity and higher TPSA value (Table 4).

### 3.3. Pharmacokinetics and Drug-likeness

Both compounds demonstrate high gastrointestinal absorption potential, supporting their suitability for oral administration. Neither compound is predicted to cross the blood–brain barrier, which may be advantageous for avoiding CNS-related side effects. A notable difference is that butyrolactone III is predicted to be a P-glycoprotein substrate, which could affect its bioavailability through efflux mechanisms. Both compounds show potential inhibition of CYP2C9 and CYP3A4, which should be considered for possible drug–drug interactions (Table 5).

Both compounds fully comply with Lipinski’s Rule of Five and other drug-likeness filters (Ghose, Veber, Egan, Muegge), with zero violations across all criteria. The bioavailability score of 0.55 for both compounds indicates a good probability of oral bioavailability. No PAINS (Pan-Assay Interference Compounds) alerts were detected, suggesting low risk of nonspecific binding in biological assays. The synthetic accessibility scores (4.29 and 4.60 on a scale of 1–10, where 1 is easiest) indicate moderate synthetic complexity (Table 6).

### 3.4. Toxicity Profile

Toxicity predictions were performed using ProTox-3.0. Both compounds were assigned to Toxicity Class 4 with a predicted LD_50_ of 2000 mg/kg (oral, rat), indicating relatively low acute toxicity. The prediction accuracy was 54.26% with average similarity to reference compounds of 46.3% and 46.87% for butyrolactone I and III, respectively (Table 7). Both compounds show favorable safety profiles regarding hepatotoxicity, neurotoxicity, and cardiotoxicity with high probability scores for inactive status. However, low-probability predictions for nephrotoxicity and respiratory toxicity were flagged as potentially active, though with marginal confidence (probability scores near 0.5).

Both compounds are predicted to be non-carcinogenic and non-mutagenic with reasonable confidence. The immunotoxicity prediction shows a marginally active status for both compounds, with butyrolactone III having a slightly higher probability (0.65). Cytotoxicity predictions indicate both compounds are unlikely to exhibit nonspecific cytotoxic effects (Table 8).

Both compounds demonstrate excellent safety profiles regarding nuclear receptor interactions. High confidence predictions indicate no interference with key endocrine signaling pathways including androgen receptor, estrogen receptor, and aryl hydrocarbon receptor. This suggests low potential for endocrine disruption (Table 9).

Both compounds show potential activity toward mitochondrial membrane potential disruption, though with moderate confidence. They are predicted to be inactive for genotoxic stress (p53) and DNA damage (ATAD5) pathways with high confidence, supporting their non-genotoxic profile (Table 10).

CYP2C9 metabolism is predicted to be active for both compounds with moderate probability, consistent with SwissADME predictions. Notably, CYP1A2 and CYP2E1 show high confidence for inactive status, while CYP3A4 shows borderline predictions with ProTox-3.0 compared to the active inhibition predicted by SwissADME (Table 11).

### 3.5. Quantum Chemical Analysis of Frontier Molecular Orbitals

Density functional theory (DFT) calculations were performed using the ORCA quantum chemistry program package to analyze the frontier molecular orbitals (FMOs) of both butyrolactone derivatives. The HOMO (Highest Occupied Molecular Orbital) and LUMO (Lowest Unoccupied Molecular Orbital) energies provide valuable information about the electronic properties and chemical reactivity of the compounds. Both compounds exhibit nearly identical HOMO energies (−6.054 and −6.059 eV), indicating similar electron-donating capabilities. The HOMO-LUMO energy gap (ΔE), which is an important indicator of molecular stability and chemical reactivity, is 4.443 eV for butyrolactone I and 4.662 eV for butyrolactone III. The larger gap observed for butyrolactone III suggests slightly higher kinetic stability and lower chemical reactivity compared to butyrolactone I. According to frontier molecular orbital theory, molecules with smaller HOMO-LUMO gaps are generally more polarizable and chemically reactive. The higher chemical hardness value for butyrolactone III (η = 2.331 eV) compared to butyrolactone I (η = 2.222 eV) indicates greater resistance to charge transfer and electronic deformation. Conversely, butyrolactone I shows a higher electrophilicity index (ω = 3.305 eV vs. 2.981 eV), suggesting a greater propensity to accept electrons from nucleophilic species. These differences may be attributed to the structural variation between the prenyl group in butyrolactone I and the epoxide moiety in butyrolactone III, which affects the electronic distribution across the molecular framework (Table 12).

The global reactivity descriptors were calculated using Koopmans’ theorem: ionization potential (I ≈ −E^HOMO^), electron affinity (A ≈ −E^LUMO^), electronegativity (χ = (I + A)/2), chemical hardness (η = (I − A)/2), chemical softness (S = 1/2η), and electrophilicity index (ω = χ^2^/2η).

**Figure 2:**
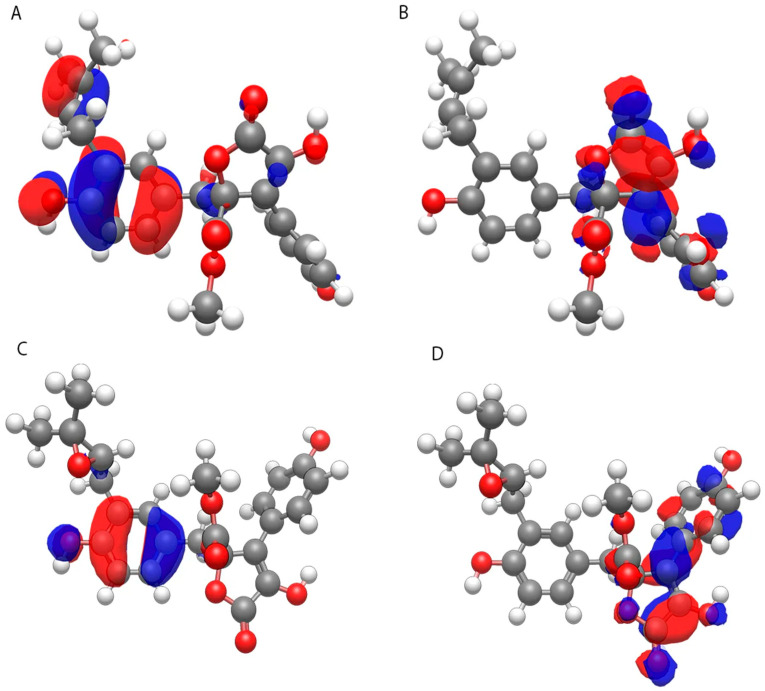
Visualization of frontier molecular orbitals: (**A**) HOMO of butyrolactone I, (**B**) LUMO of butyrolactone I, (**C**) HOMO of butyrolactone III, (**D**) LUMO of butyrolactone III. Blue and red surfaces represent positive and negative phases of the molecular orbitals, respectively.

The spatial distribution of frontier molecular orbitals (Figure 2) provides insight into the potential reactive sites of both compounds. In both molecules, the HOMO electron density is predominantly localized on the hydroxyphenyl ring and the prenyl/epoxide substituent, indicating these regions as primary electron-donating sites. The LUMO electron density is mainly distributed over the butyrolactone core and the p-hydroxyphenyl moiety, suggesting these areas as potential electron-accepting sites during electrophilic interactions.

### 3.6. Molecular Docking Studies

**Figure 3:**
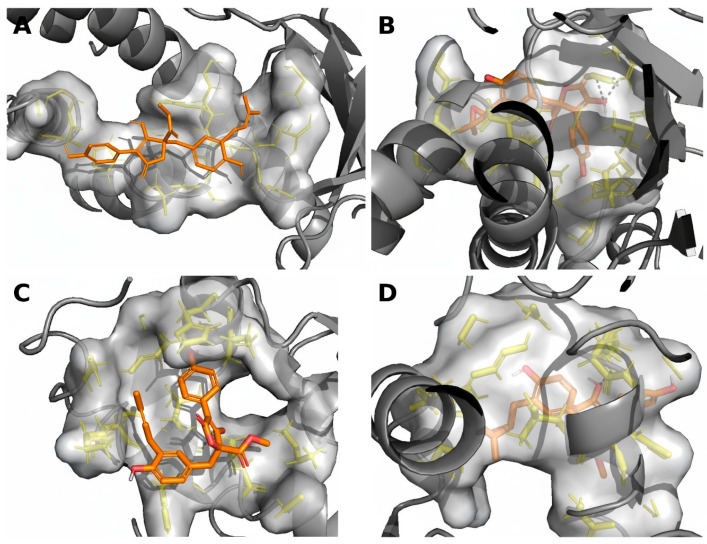
Molecular docking poses of butyrolactone derivatives in the active sites of therapeutic targets. (**A**) butyrolactone I in human acetylcholinesterase (AChE, PDB: 4EY7), binding affinity: −8.8 kcal/mol; (**B**) butyrolactone III in AChE, binding affinity: −9.0 kcal/mol; (**C**) butyrolactone I in human MDM2 (PDB: 4HG7), binding affinity: −7.8 kcal/mol; (**D**) butyrolactone III in MDM2, binding affinity: −7.7 kcal/mol. Ligands are shown as orange sticks, binding site residues as yellow sticks, and protein backbone as gray cartoon. The binding pocket surface is displayed in transparent gray. Hydrogen bonds are indicated as black dashed lines.

**Figure 4:**
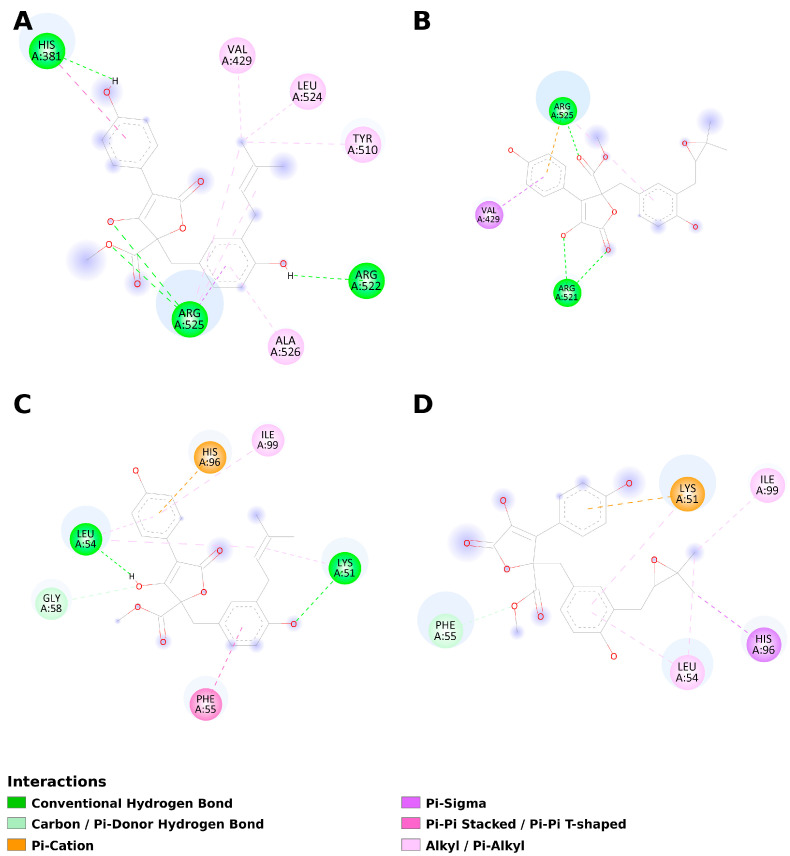
Two-dimensional interaction diagrams of butyrolactone derivatives with therapeutic targets generated using Discovery Studio Visualizer. (**A**) butyrolactone I–AChE interactions; (**B**) butyrolactone III–AChE interactions; (**C**) butyrolactone I–MDM2 interactions; (**D**) butyrolactone III–MDM2 interactions.

The binding affinities (kcal/mol) obtained from molecular docking are summarized in Table 13. More negative values indicate stronger predicted binding affinity. The best docking poses of butyrolactone I and butyrolactone III in the active sites of AChE and MDM2 are visualized in Figure 3, while the detailed 2D interaction diagrams are presented in Figure 4.

Both butyrolactone derivatives demonstrated the strongest binding affinity toward acetylcholinesterase (AChE), with butyrolactone III (−9.0 kcal/mol) showing better binding than butyrolactone I (−8.8 kcal/mol). Notably, butyrolactone III exhibited binding affinity comparable to the reference drug donepezil (−8.3 kcal/mol), a clinically approved AChE inhibitor used in Alzheimer’s disease therapy. These results suggest that butyrolactone derivatives may serve as promising scaffolds for the development of novel cholinesterase inhibitors. Because the docking grid was centered on the co-crystallized donepezil position, both butyrolactones dock into the same active-site gorge as donepezil, a cavity lined by catalytic- and peripheral-anionic-site residues including Trp86 and Trp286, supporting an overall binding mode comparable to that of the reference inhibitor.

MDM2 showed the second-best binding affinities, with butyrolactone I (−7.8 kcal/mol) demonstrating binding affinity slightly exceeding the reference compound nutlin-3a (−7.6 kcal/mol), a well-known MDM2 inhibitor. Butyrolactone III exhibited comparable affinity (−7.7 kcal/mol). These findings indicate potential anticancer activity through the p53 reactivation mechanism. Both ligands were docked into the same hydrophobic p53-binding cleft occupied by nutlin-3a; this pocket, lined by residues such as Leu54, Gly58, Ile61, Met62, and Val93 and comprising the Phe19, Trp23, and Leu26 sub-sites, supports an overall binding mode comparable to that of the reference antagonist. Both compounds exhibited notable binding to COX-2 (butyrolactone I: −7.2 kcal/mol; butyrolactone III: −6.7 kcal/mol) compared to the reference drug rofecoxib (−10.2 kcal/mol), suggesting moderate anti-inflammatory activity via this pathway. Similarly, moderate binding to Topoisomerase IV (butyrolactone I: −5.6 kcal/mol; butyrolactone III: −5.2 kcal/mol) was observed, which was weaker than the reference antibiotic novobiocin (−6.3 kcal/mol), indicating moderate antibacterial potential through this target.

### 3.7. Molecular Dynamics Simulation Results

**Figure 5:**
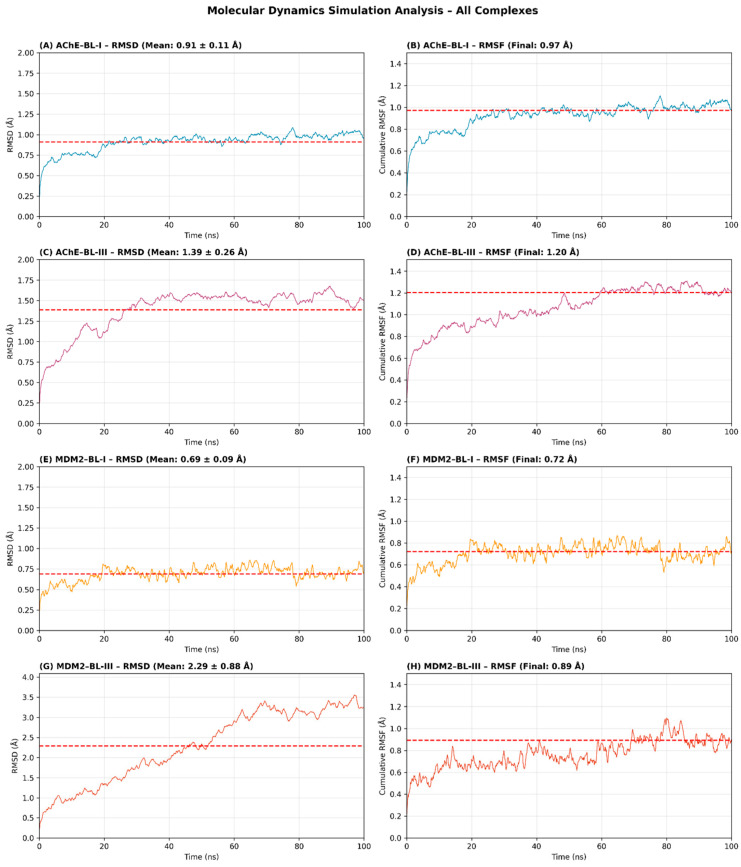
Molecular dynamics simulation analysis of butyrolactone–protein complexes over 100 ns. (**A**) Backbone RMSD of AChE–BL-I complex (mean: 0.91 ± 0.11 Å). (**B**) Cumulative RMSF of AChE–BL-I complex. (**C**) Backbone RMSD of AChE–BL-III complex (mean: 1.39 ± 0.26 Å). (**D**) Cumulative RMSF of AChE–BL-III complex. (**E**) Backbone RMSD of MDM2–BL-I complex (mean: 0.69 ± 0.09 Å). (**F**) Cumulative RMSF of MDM2–BL-I complex. (**G**) Backbone RMSD of MDM2–BL-III complex (mean: 2.29 ± 0.88 Å). (**H**) Cumulative RMSF of MDM2–BL-III complex. Red dashed lines indicate mean values. All simulations were performed at 310 K under NPT conditions. The MDM2–BL-III complex exhibited significant instability with RMSD values exceeding 3.5 Å, indicating that BL-III does not form a stable complex with MDM2. In each panel, the red dashed horizontal line indicates the reference level: the mean backbone RMSD in the RMSD panels (**A**,**C**,**E**,**G**) and the final converged cumulative RMSF value in the RMSF panels (**B**,**D**,**F**,**H**).

To validate the molecular docking results and assess the dynamic stability of the predicted binding modes, 100 ns MD simulations were performed for all four protein–ligand complexes: AChE–BL-I, AChE–BL-III, MDM2–BL-I, and MDM2–BL-III. The backbone RMSD profiles and simulation statistics are summarized in Table 14 and Figure 5.

Both AChE complexes demonstrated excellent structural stability throughout the 100 ns simulation period. The AChE–BL-I complex exhibited a mean backbone RMSD of 0.91 ± 0.11 Å, with values remaining consistently below 1.1 Å after initial equilibration (Figure 5A). This low RMSD indicates that BL-I binding did not induce significant conformational changes in the enzyme structure, and the ligand maintained stable interactions within the active site gorge. The AChE–BL-III complex showed slightly higher but still acceptable RMSD values (mean: 1.39 ± 0.26 Å, maximum: 1.68 Å) (Figure 5C). The RMSD profile displayed a gradual increase during the first 40 ns followed by stabilization, suggesting the complex reached a stable equilibrium conformation. Both AChE complexes remained well below the 2.0 Å threshold typically considered indicative of stable protein–ligand binding, confirming that the docking-predicted binding modes are dynamically viable.

The MDM2 complexes exhibited contrasting stability profiles. The MDM2–BL-I complex was remarkably stable, with the lowest mean RMSD among all complexes (0.69 ± 0.09 Å) and maximum values never exceeding 0.86 Å (Figure 5E). This exceptional stability is consistent with the favorable binding affinity predicted by docking (−7.8 kcal/mol, slightly exceeding the reference inhibitor nutlin-3a) and suggests tight complementarity between BL-I and the MDM2 p53-binding pocket. In contrast, the MDM2–BL-III complex showed significant instability, with RMSD values reaching up to 3.55 Å and exhibiting large fluctuations throughout the simulation (Figure 5G). The mean RMSD of 2.29 ± 0.88 Å and the high standard deviation indicate that the complex did not achieve a stable conformation. This instability correlates with the docking score of BL-III for MDM2 (−7.7 kcal/mol vs. −7.8 kcal/mol for BL-I) and suggests that the structural differences between BL-I and BL-III significantly impact binding to MDM2.

The cumulative root mean square fluctuation (RMSF) profiles were monitored throughout the 100 ns simulations to assess the convergence of residue flexibility and overall structural dynamics of the protein–ligand complexes (Figure 5B,D,F,H). The cumulative RMSF represents the time-averaged atomic fluctuations and provides insight into whether the simulations achieved adequate sampling and equilibration.

For the AChE complexes, the AChE–BL-I system exhibited a final cumulative RMSF of 0.97 Å (Figure 5B), with values stabilizing after approximately 40 ns of simulation. The AChE–BL-III complex showed a slightly higher final RMSF of 1.20 Å (Figure 5D), consistent with the marginally elevated RMSD values observed for this system. Both AChE complexes demonstrated good convergence behavior, with the cumulative RMSF profiles plateauing in the latter half of the simulation, indicating that sufficient conformational sampling was achieved. The relatively low RMSF values for both complexes suggest that ligand binding stabilizes the overall protein structure without inducing excessive local fluctuations.

The MDM2 complexes exhibited distinct flexibility patterns that correlated with their binding stability. The MDM2–BL-I complex displayed the lowest cumulative RMSF among all systems (final value: 0.72 Å; Figure 5F), reflecting the exceptional structural rigidity of this complex. This low flexibility is consistent with the tight binding observed in both docking (−7.8 kcal/mol) and RMSD analysis (mean: 0.69 ± 0.09 Å), suggesting that BL-I effectively restricts conformational dynamics of the MDM2 p53-binding pocket. In contrast, the MDM2–BL-III complex showed a higher final RMSF of 0.89 Å (Figure 5H), with greater fluctuations throughout the simulation trajectory. Although this value remains relatively low, the increased flexibility compared to MDM2–BL-I correlates with the unstable RMSD profile observed for this complex and further supports the conclusion that the epoxide moiety of BL-III provides less effective stabilization of the MDM2 binding site than the prenyl group of BL-I.

## 4. Discussion

In silico methodologies have become indispensable tools in contemporary drug discovery, enabling rapid and cost-effective preliminary evaluation of prospective pharmacological candidates [51]. The present investigation comprehensively characterized the pharmacokinetic, toxicological, electronic, and binding properties of butyrolactone I and butyrolactone III, two structurally related γ-butyrolactone derivatives isolated from the marine-derived fungus *A. terreus*.

The computational predictions presented in this study show good qualitative concordance with published experimental data, lending indirect support to the in silico predictions while not, in themselves, constituting direct experimental validation of the specific targets examined. Comprehensive in vitro characterization of butyrolactone I and butyrolactone III by Uras et al. (2021, 2022) [52,53] revealed striking differences in their pharmacological profiles that directly correlate with our current computational findings.

Experimental studies demonstrated that butyrolactone I exhibited pronounced inhibitory effects on elastase release from human neutrophils with an IC_50_ of 2.30 μM, exceeding the efficacy of the reference compound genistein. The compound showed no evidence of toxicity at concentrations up to 10 μM and exhibited moderate anti-allergic activity in RBL-2H3 mast cells. In the *A. terreus* study [52], butyrolactone I was active whereas butyrolactone III was inactive. In the subsequent *A. costaricaensis* study [53], butyrolactone I remained active, while an oxirane-bearing butenolide (butyrolactone V) was inactive, further supporting the adverse impact of epoxidation/oxirane motifs on activity in these assays. Specifically, butyrolactone III showed no anti-inflammatory activity in the elastase-release assay (IC_50_ > 10 µM) and was inactive in RBL-2H3 degranulation assays measured by β-hexosaminidase release (IC_50_ > 100 µM) [52].

These experimental findings are broadly consistent with our computational predictions. Our current study extends them by demonstrating that the structure-activity relationship observed experimentally generalizes computationally to other therapeutic targets. The molecular dynamics simulations reveal that the MDM2-butyrolactone I complex exhibits exceptional stability (mean RMSD: 0.69 ± 0.09 Å), while the MDM2-butyrolactone III complex shows significant instability (mean RMSD: 2.29 ± 0.88 Å, reaching 3.55 Å). This computational observation parallels the experimental finding that structural modification from prenyl to epoxide results in loss of biological activity in these assays. It should be emphasized, however, that the elastase-release and degranulation assays report anti-inflammatory phenotypes rather than MDM2 engagement; they therefore corroborate the general prenyl-versus-epoxide structure–activity trend captured by our simulations, but do not directly validate the proposed MDM2 binding mechanism, which still requires dedicated biochemical testing.

The predictive validity of our computational approach is further supported by multiple independent experimental studies on butyrolactone I and structurally related *A. terreus* metabolites. The anticancer potential of butyrolactone I predicted through MDM2 binding is consistent with extensive experimental evidence demonstrating its cytotoxic and cell cycle regulatory effects. Kitagawa et al. (1993) [16] established butyrolactone I as a selective inhibitor of CDK2 and CDC2 kinases, key regulators of cell cycle progression. Subsequent studies by Nishio et al. (1996) [17] demonstrated antitumor effects on human lung cancer cell lines, while Ghfar et al. (2021) [19] showed induction of apoptosis in prostate and ovarian cancer cells. More recently, Ahn et al. (2020) [18] revealed through X-ray crystallographic analysis that butyrolactone I functions as both a CDK5 inhibitor and a partial agonist of peroxisome proliferator-activated receptor γ (PPARγ), demonstrating experimentally validated polypharmacological activity. The convergence of CDK inhibition, p53 pathway modulation via MDM2, and PPARγ agonism suggests butyrolactone I functions as a multi-target anticancer agent, consistent with our computational predictions of broad therapeutic potential.

For acetylcholinesterase inhibition, our computational prediction that butyrolactone III exhibits binding affinity exceeding donepezil (−9.0 vs. −8.3 kcal/mol) is supported by experimental studies on structurally related A. terreus metabolites. Nong et al. (2014) [54] demonstrated that territrem derivatives from the same fungal source exhibited potent acetylcholinesterase inhibition with IC_50_ values of approximately 4.2–4.5 nM, confirming that A. terreus produces highly effective AChE inhibitors. Because territrems are a chemically distinct class of A. terreus metabolites, this evidence provides only indirect support, rather than direct validation, for our computational prediction that butyrolactones may represent promising scaffolds for neuroprotective agents. Direct experimental evidence for butyrolactone I neuroprotective activity was provided by Zhang et al. (2018) [55], who demonstrated significant anti-neuroinflammatory effects in LPS-stimulated BV-2 microglia cells through suppression of the NF-κB pathway, reduction of nitric oxide and pro-inflammatory cytokine production, and inhibition of iNOS and COX-2 expression. These findings are consistent with, but do not directly confirm, our computational prediction that butyrolactones may represent promising scaffolds for neuroprotective agents.

The qualitative consistency between published in vitro data and our in silico predictions lends credibility to the computational workflow employed and to the biological relevance of the identified structure–activity relationships, although it does not amount to direct experimental validation of the individual docking and molecular-dynamics predictions. This concordance reinforces that the prenyl group in butyrolactone I is critical for target engagement across multiple protein families, while the epoxide ring in butyrolactone III compromises binding stability and eliminates biological activity in certain targets while potentially enhancing interactions with others, such as acetylcholinesterase.

Because compound isolation is often time-consuming, in silico approaches can rationally prioritize candidates for downstream testing, saving both time and reagents. Previous studies analyzed butenolide derivatives isolated from marine Aspergillus species for their anti-inflammatory, anti-allergic, and potential antiviral properties. Comprehensive in vitro and in silico characterization by Uras et al. (2021) focused on metabolites from *A. terreus* [52], while Uras et al. (2022) investigated compounds from *A. costaricaensis* [53]. These complementary studies enabled assessment of consistency between computational predictions and experimental validation across different fungal species producing structurally related metabolites.

The physicochemical profiling revealed that both butyrolactone derivatives possess favorable drug-like characteristics, demonstrating complete compliance with Lipinski’s Rule of Five and additional pharmaceutical filters including Ghose, Veber, Egan, and Muegge criteria. The topological polar surface area (TPSA) values of 113.29 Å^2^ and 125.82 Å^2^ suggest moderate membrane permeability while remaining below the threshold typically associated with poor oral absorption [32,34]. Notably, the structural distinction between the two compounds (the prenyl moiety in butyrolactone I versus the epoxide group in butyrolactone III) manifests in differential lipophilicity profiles, with butyrolactone III exhibiting enhanced aqueous solubility attributable to its more polar epoxide functionality.

Regarding pharmacokinetics, both butyrolactones demonstrate high predicted gastrointestinal absorption, supporting their suitability for oral administration. Although our in silico workflow predicted limited blood–brain barrier penetration, peripheral mechanisms (e.g., via the gut–brain axis) may also influence neurodegenerative pathology [56]. The prediction that butyrolactone III serves as a P-glycoprotein substrate warrants consideration in future formulation strategies, as P-gp-mediated efflux can significantly attenuate bioavailability [57]. The identified CYP2C9 and CYP3A4 inhibitory potential of both compounds necessitates careful evaluation of drug–drug interaction profiles in subsequent preclinical development.

The toxicological profiling revealed reassuring safety margins for both derivatives. The assignment to Toxicity Class 4 with predicted LD_50_ values of 2000 mg/kg indicates relatively low acute oral toxicity. The high-confidence predictions of inactivity toward hepatotoxicity, neurotoxicity, and cardiotoxicity endpoints are particularly encouraging, as safety/toxicology concerns are among the major causes of drug attrition in clinical development [58]. Furthermore, in silico toxicity profiling did not flag strong activity across common endocrine-disruption–relevant nuclear receptors; however, experimental data indicate that butyrolactone I can act as a partial agonist of PPARγ [18], consistent with polypharmacology rather than an absence of nuclear receptor engagement.

The frontier molecular orbital analysis revealed nearly identical HOMO energies for both compounds (−6.054 and −6.059 eV), indicating comparable electron-donating capabilities. However, the HOMO–LUMO energy gaps (4.443 eV for butyrolactone I versus 4.662 eV for butyrolactone III) suggest distinct reactivity profiles. According to frontier molecular orbital theory, the larger energy gap observed for butyrolactone III correlates with enhanced kinetic stability and reduced chemical reactivity [59,60], which may translate to improved metabolic resistance in vivo. Conversely, the higher electrophilicity index of butyrolactone I (ω = 3.305 eV versus 2.981 eV) suggests a greater propensity for electrophilic interactions with nucleophilic biological targets.

The molecular docking studies revealed compelling binding affinities toward acetylcholinesterase (AChE), with butyrolactone III (−9.0 kcal/mol) demonstrating binding affinity exceeding the clinically approved reference drug donepezil (−8.3 kcal/mol) and higher than butyrolactone I (−8.8 kcal/mol). These results suggest that butyrolactone derivatives are promising AChE inhibitor scaffolds. These predictions are consistent with reports of potent AChE inhibition by structurally related A. terreus metabolites, including territrem derivatives with low-nanomolar IC_50_ values [54], and with favorable predicted AChE binding for butyrolactone I reported by Ma et al. (2025) [61].

The docking analysis against the MDM2-p53 interaction interface yielded particularly noteworthy results. Butyrolactone I demonstrated binding affinity (−7.8 kcal/mol) slightly exceeding nutlin-3a, a well-characterized small-molecule MDM2 antagonist (IC_50_ ≈ 90 nM for disrupting the MDM2–p53 interaction [62]). Nutlin-3 is a prototypical MDM2 antagonist, and more drug-like derivatives (e.g., RG7112) have advanced into Phase I clinical trials [63,64]. However, it is important to note that numerically equivalent docking scores do not necessarily translate to equivalent biological activity, as factors such as binding kinetics, induced-fit effects, and cellular permeability are not captured by rigid-receptor docking. Biochemical validation through MDM2-p53 disruption assays is required to confirm the therapeutic potential of butyrolactone I. The MDM2-p53 interaction represents a validated oncological target, as MDM2 overexpression in tumors harboring wild-type p53 effectively suppresses the tumor suppressor function [64]. The anticancer potential of butyrolactone I has been previously established through multiple mechanisms, including selective inhibition of cyclin-dependent kinases CDK1/cyclin B (IC_50_ = 0.65 μM), CDK2/cyclin A (IC_50_ = 1.38 μM), CDK2/cyclin E (IC_50_ = 0.66 μM), and potent CDK5 inhibition (IC_50_: 0.17 μM for CDK5/p25 and 0.22 μM for CDK5/p35). Notably, Ahn and colleagues (2020) demonstrated that butyrolactone I exhibits polypharmacological activity, functioning simultaneously as a CDK5 inhibitor and a partial agonist of peroxisome proliferator-activated receptor γ (PPARγ), as confirmed by X-ray crystallographic analysis [18].

The molecular dynamics simulations provided crucial validation of the docking-predicted binding modes. The exceptional stability of the AChE–butyrolactone complexes, characterized by backbone RMSD values remaining below 2.0 Å throughout the entire 100 ns trajectory, confirms the feasibility of these interactions under physiological conditions. The AChE–BL-I complex exhibited particularly favorable stability metrics (mean RMSD: 0.91 ± 0.11 Å), indicating minimal perturbation to enzyme architecture upon ligand binding. Similarly, the MDM2–BL-I complex demonstrated remarkable stability (mean RMSD: 0.69 ± 0.09 Å), corroborating the favorable docking-predicted binding affinity. In contrast, the pronounced instability of the MDM2–BL-III complex (mean RMSD: 2.29 ± 0.88 Å, reaching 3.55 Å) reveals that the epoxide moiety replacing the prenyl group significantly compromises MDM2 binding, highlighting the critical role of this structural element in MDM2 recognition.

Although the four targets were selected to represent distinct therapeutic areas, they are not biologically independent, and several converge on shared pathological processes that provide a coherent rationale for a multi-target profile. Chronic inflammation constitutes a common mechanistic thread, as COX-2 is a central effector of inflammatory signaling that is also frequently up-regulated in tumors and in the neuroinflammatory environment of Alzheimer’s disease, where microglial COX-2 induction accompanies cholinergic decline. The MDM2-p53 axis is reciprocally linked to this inflammatory signaling, since p53 restrains NF-κB-driven COX-2 expression, so a scaffold engaging both COX-2 and the MDM2-p53 interface could act on intersecting nodes of tumor-associated inflammation. Neurodegeneration similarly integrates cholinergic dysfunction (AChE), neuroinflammation (COX-2/NF-κB), and oxidative stress, processes that have all been reported for butyrolactone I in microglial and in vivo models [55,56,65,66]. In contrast, the antibacterial target topoisomerase IV lies on a largely independent axis and is best interpreted as a separate, weaker activity rather than part of this inflammatory, oncological, and neurological network. Taken together, the predicted engagement of COX-2, MDM2, and AChE is consistent with a polypharmacological profile directed at overlapping inflammatory and cell-survival pathways, whereas the divergent behavior of butyrolactones I and III across these targets indicates that any multi-target application would be compound- and target-selective rather than uniform.

Other experimentally or computationally reported targets of these and related metabolites, such as cyclin-dependent kinases and PPARγ [16,18], human topoisomerase II [67], butyrylcholinesterase [68], protein tyrosine phosphatase 1B (PTP1B) [69] and α-glucosidase [70], as well as the SARS-CoV-2 main protease (Mpro) [52,53], were deliberately not docked in the present panel. Cyclin-dependent kinases and PPARγ are already experimentally established for butyrolactone I and are therefore treated as orthogonal, literature-based reference points rather than docking targets; the reported topoisomerase II activity is represented at the family level by the bacterial type II topoisomerase (topoisomerase IV) retained here as the antibacterial exemplar; butyrylcholinesterase is closely related to the acetylcholinesterase target already included; and the metabolic targets PTP1B and α-glucosidase, together with Mpro, fall outside the four therapeutic areas prioritized in this study.

Butyrolactone I shows anti-neuroinflammatory activity in BV-2 microglia and mitigates cognitive deficits in an in vivo zebrafish model [55,56], supporting its neuroprotective potential. Additional studies indicate broader cytoprotective/anti-apoptotic activity under oxidative/ER stress conditions [65], while oxidative stress is a key pathological component of Alzheimer’s disease [66]. The comparative analysis of butyrolactone I and III provides valuable structure-activity relationship insights. The prenyl moiety of butyrolactone I contributes to enhanced lipophilicity, potentially facilitating membrane permeation and hydrophobic binding pocket interactions, as evidenced by its superior MDM2 binding stability. Conversely, the epoxide functionality of butyrolactone III introduces additional hydrogen bonding capacity and increased polarity, which may explain its enhanced aqueous solubility and marginally improved AChE binding affinity. For cholinesterase inhibition, our docking results suggest superior binding affinity of butyrolactone III relative to donepezil (−9.0 vs. −8.3 kcal/mol). While docking scores provide valuable preliminary insights, their inherent uncertainty necessitates experimental validation [71], although it should be noted that territrem derivatives from the same fungal source exhibited markedly higher potency with IC_50_ values of approximately 4.2–4.5 nM [54].

Several limitations warrant acknowledgment. Computational predictions require experimental validation across biochemical, cellular, and whole-organism models. The discordance between ProTox-3.0 and SwissADME predictions for certain CYP450 interactions highlights the inherent uncertainty in computational toxicology estimates. This uncertainty is underscored by the modest internal confidence of the toxicity model itself: the ProTox-3.0 acute-toxicity prediction was returned with an accuracy of only 54.26% and an average similarity to reference compounds of approximately 46%, indicating that both butyrolactones fall in a sparsely populated region of the model’s applicability domain. The favorable organ-toxicity and endocrine-disruption predictions should therefore be regarded as low-confidence, hypothesis-level indicators rather than definitive safety assessments, particularly for the several endpoints (nephrotoxicity, respiratory toxicity, mitochondrial membrane potential, and immunotoxicity) flagged with borderline probabilities near 0.5. Accordingly, the ADMET and toxicity profiles reported here should be confirmed with orthogonal predictive tools and, ultimately, by direct experimental measurement before any safety conclusions are drawn. Furthermore, the molecular docking approach does not fully account for protein flexibility, solvation effects, or entropic contributions to binding free energy [71]. Although molecular dynamics simulations address some of these limitations, extended simulation timescales may be required to capture slower conformational transitions relevant to binding kinetics. Future investigations should prioritize in vitro enzyme inhibition assays, cellular efficacy studies in relevant disease models, and pharmacokinetic characterization in appropriate animal models. In addition, the ADMET and toxicity endpoints were derived from a limited number of predictive servers (SwissADME and ProTox-3.0); convergent predictions from orthogonal tools and, ultimately, experimental measurement would be required before firm conclusions about absorption, metabolism, and safety can be drawn. The docking panel was intentionally restricted to four representative targets and did not include the cyclin-dependent kinases that are the experimentally established targets of butyrolactone I, so the analysis should be regarded as a hypothesis-generating exploration of new target space rather than a comprehensive target profile. The findings also pertain specifically to butyrolactones I and III and should not be extrapolated uncritically to other butyrolactone or γ-butyrolactone derivatives without dedicated analysis. A rational translational pipeline emerging from this work would proceed from in vitro target-engagement and enzyme-inhibition assays (AChE inhibition; MDM2–p53 disruption; COX-2 and topoisomerase IV activity), through cellular efficacy and selectivity profiling in disease-relevant models, to ADME/PK characterization and preliminary in vivo efficacy and safety studies for the most promising scaffold.

## 5. Conclusions

This in silico study identifies butyrolactone I and butyrolactone III as promising natural product scaffolds with distinct therapeutic profiles. Both compounds demonstrate excellent drug-like properties, complete Lipinski compliance, high predicted gastrointestinal absorption, and favorable safety profiles (toxicity class 4, no hepatotoxic, neurotoxic, cardiotoxic, carcinogenic, or mutagenic liabilities). The predicted CYP2C9 and CYP3A4 inhibition warrants evaluation of potential drug–drug interactions in future studies. Quantum chemical analysis revealed that butyrolactone III exhibits enhanced kinetic stability (HOMO–LUMO gap: 4.662 eV vs. 4.443 eV), suggesting potentially improved metabolic resistance. Molecular docking identified target-selective binding profiles: butyrolactone III demonstrated acetylcholinesterase binding affinity (−9.0 kcal/mol) exceeding donepezil (−8.3 kcal/mol), while butyrolactone I showed MDM2 binding slightly exceeding nutlin-3a (−7.8 kcal/mol). Molecular dynamics simulations (100 ns) supported these predictions, revealing stable AChE complexes (RMSD < 2.0 Å) and exceptional MDM2–butyrolactone I stability (RMSD: 0.69 ± 0.09 Å), while the MDM2–butyrolactone III complex exhibited significant instability (RMSD up to 3.55 Å), highlighting the critical role of the prenyl group in MDM2 recognition.

These findings, which are consistent with, though not a direct experimental validation of, previously published in vitro data, support the further evaluation of butyrolactone III as a scaffold for neuroprotective agents and butyrolactone I as a p53 pathway modulator for cancer therapy. Future in vitro and in vivo studies are necessary to validate these computational predictions and assess pharmacokinetic properties, metabolic stability, and therapeutic efficacy in relevant disease models.

## Figures and Tables

**Table 1:** Target proteins selected for molecular docking studies.

Target	PDB ID	Organism	Reference Ligand	Application
COX-2	5KIR	*H. sapiens*	Rofecoxib	Anti-inflammatory
Topo IV	1S14	*E. coli*	Novobiocin	Antibacterial
MDM2	4HG7	*H. sapiens*	Nutlin-3a	Anticancer
AChE	4EY7	*H. sapiens*	Donepezil	Neuroprotective

**Table 2:** Physicochemical properties of butyrolactone I and butyrolactone III.

Parameter	Butyrolactone I	Butyrolactone III
Molecular Weight (g/mol)	424.44	440.44
Number of Heavy Atoms	31	32
Number of Aromatic Heavy Atoms	12	12
Fraction Csp^3^	0.25	0.33
Number of Rotatable Bonds	7	7
Number of H-bond Acceptors	7	8
Number of H-bond Donors	3	3
Molar Refractivity	114.89	114.37
TPSA (Å^2^)	113.29	125.82

**Table 3:** Lipophilicity parameters.

Parameter	Butyrolactone I	Butyrolactone III
iLOGP	2.89	2.79
XLOGP3	4.37	3.01
WLOGP	3.59	2.80
MLOGP	2.02	1.30
Silicos-IT Log *p*	4.19	4.01
Consensus Log *p*	3.41	2.78

**Table 4:** Aqueous solubility predictions.

Parameter	Butyrolactone I	Butyrolactone III
ESOL Log S	−5.05	−4.28
ESOL Class	Moderately soluble	Moderately soluble
Ali Log S	−6.47	−5.32
Ali Class	Poorly soluble	Moderately soluble
Silicos-IT Log Sw	−5.54	−5.48
Silicos-IT Class	Moderately soluble	Moderately soluble

**Table 5:** Pharmacokinetic predictions.

Parameter	Butyrolactone I	Butyrolactone III
GI Absorption	High	High
BBB Permeant	No	No
P-gp Substrate	No	Yes
CYP1A2 Inhibitor	No	No
CYP2C19 Inhibitor	No	No
CYP2C9 Inhibitor	Yes	Yes
CYP2D6 Inhibitor	No	No
CYP3A4 Inhibitor	Yes	Yes
Log Kp (cm/s)	−5.79	−6.85

**Table 6:** Drug-likeness evaluation.

Parameter	Butyrolactone I	Butyrolactone III
Lipinski Violations	0	0
Ghose Violations	0	0
Veber Violations	0	0
Egan Violations	0	0
Muegge Violations	0	0
Bioavailability Score	0.55	0.55
PAINS Alerts	0	0
Brenk Alerts	2	2
Synthetic Accessibility	4.29	4.60

**Table 7:** Organ toxicity predictions.

Target	Butyrolactone I	Butyrolactone III
Hepatotoxicity	Inactive (0.70)	Inactive (0.72)
Neurotoxicity	Inactive (0.83)	Inactive (0.84)
Nephrotoxicity	Active (0.55)	Active (0.53)
Respiratory Toxicity	Active (0.52)	Active (0.57)
Cardiotoxicity	Inactive (0.77)	Inactive (0.72)

**Table 8:** Toxicity endpoints.

Endpoint	Butyrolactone I	Butyrolactone III
Carcinogenicity	Inactive (0.58)	Inactive (0.54)
Immunotoxicity	Active (0.54)	Active (0.65)
Mutagenicity	Inactive (0.70)	Inactive (0.59)
Cytotoxicity	Inactive (0.75)	Inactive (0.63)
BBB	Active (0.54)	Active (0.56)
Ecotoxicity	Inactive (0.60)	Inactive (0.59)
Clinical Toxicity	Active (0.55)	Active (0.55)
Nutritional Toxicity	Active (0.68)	Active (0.70)

**Table 9:** Tox21 Nuclear receptor signaling pathway predictions.

Receptor	Butyrolactone I	Butyrolactone III
Aryl hydrocarbon Receptor (AhR)	Inactive (0.87)	Inactive (0.87)
Androgen Receptor (AR)	Inactive (0.92)	Inactive (0.91)
AR Ligand Binding Domain	Inactive (0.92)	Inactive (0.92)
Aromatase	Inactive (0.78)	Inactive (0.81)
Estrogen Receptor Alpha (ER)	Inactive (0.77)	Inactive (0.80)
ER Ligand Binding Domain	Inactive (0.93)	Inactive (0.96)
PPAR-Gamma	Inactive (0.77)	Inactive (0.78)

**Table 10:** Tox21 Stress response pathway predictions.

Pathway	Butyrolactone I	Butyrolactone III
nrf2/ARE	Inactive (0.75)	Inactive (0.74)
Heat Shock Element (HSE)	Inactive (0.75)	Inactive (0.74)
Mitochondrial Membrane Potential	Active (0.60)	Active (0.55)
Tumor Suppressor p53	Inactive (0.52)	Inactive (0.55)
ATAD5	Inactive (0.92)	Inactive (0.91)

**Table 11:** Cytochrome P450 metabolism predictions (ProTox-3.0).

Enzyme	Butyrolactone I	Butyrolactone III
CYP1A2	Inactive (0.90)	Inactive (0.92)
CYP2C19	Inactive (0.51)	Inactive (0.53)
CYP2C9	Active (0.74)	Active (0.65)
CYP2D6	Inactive (0.74)	Inactive (0.74)
CYP3A4	Inactive (0.56)	Inactive (0.50)
CYP2E1	Inactive (0.99)	Inactive (0.99)

**Table 12:** Frontier molecular orbital energies and global reactivity descriptors.

Parameter	Butyrolactone I	Butyrolactone III
E^HOMO^ (eV)	−6.054	−6.059
E^LUMO^ (eV)	−1.611	−1.397
ΔE (HOMO-LUMO gap) (eV)	4.443	4.662
Ionization potential, I (eV)	6.054	6.059
Electron affinity, A (eV)	1.611	1.397
Electronegativity, χ (eV)	3.833	3.728
Chemical hardness, η (eV)	2.222	2.331
Chemical softness, S (eV^−1^)	0.225	0.214
Electrophilicity index, ω (eV)	3.305	2.981

**Table 13:** Molecular docking results: binding affinities (kcal/mol).

Target (PDB ID)	Butyrolactone I	Butyrolactone III	Positive Control
AChE (4EY7)	−8.8	−9.0	Donepezil: −8.3
MDM2 (4HG7)	−7.8	−7.7	Nutlin−3a: −7.6
Topo IV (1S14)	−5.6	−5.2	Novobiocin: −6.3
COX-2 (5KIR)	−7.2	−6.7	Rofecoxib: −10.2

**Table 14:** Summary of molecular dynamics simulation results for all protein–ligand complexes.

Complex	Mean RMSD (Å)	RMSD Range (Å)	Max RMSD (Å)	Stability
AChE–BL-I	0.91 ± 0.11	0.24–1.09	1.09	Stable
AChE–BL-III	1.39 ± 0.26	0.24–1.68	1.68	Stable
MDM2–BL-I	0.69 ± 0.09	0.23–0.86	0.86	Highly stable
MDM2–BL-III	2.29 ± 0.88	0.22–3.55	3.55	Unstable

## Data Availability

The original contributions presented in this study are included in the article. Further inquiries can be directed to the corresponding authors.
